# Postoperative Complications Following Appendectomy: A Single-Center Retrospective Study

**DOI:** 10.7759/cureus.70219

**Published:** 2024-09-25

**Authors:** Amnah A Dobel, Nawaf A Alkhaldi, Alshaima A Alkharashi, Nour H Aljamaan, Mohammad Eid M Mahfouz

**Affiliations:** 1 Department of Surgery, College of Medicine, Taif University, Taif, SAU; 2 Department of Surgery, King Faisal University, Al-Hofuf, SAU

**Keywords:** appendectomy, appendicitis, retrospective study, saudi arabia, surgical complications, taif city

## Abstract

Background

Appendicitis is a common surgical emergency with a global incidence rate of approximately 8%, necessitating prompt intervention to prevent complications. Appendectomy, either through open surgery or laparoscopy, is the standard treatment. Understanding the factors contributing to post-appendectomy complications is crucial for improving patient outcomes.

Aim

This retrospective study aimed to assess the surgical outcomes of various surgical approaches for appendicitis in Taif City, Saudi Arabia, specifically at King Abdulaziz Specialist Hospital.

Methodology

A total of 220 patients who underwent appendectomy in 2022 were included in the study. Data were obtained from medical files, and statistical analysis was performed using IBM SPSS Statistics for Windows, Version 26.0 (Released 2019; IBM Corp., Armonk, NY, USA). chi-square and Fisher’s exact tests were utilized, with a significance level set at p < 0.05.

Results

The majority of patients fell within the 20-39 age group (n = 124, 56.4%), and males constituted 63% (n = 140) of the cohort. Mean complications post-surgery were observed in 5.5% (n = 12) of cases, with surgical site infection being the most common complication (n = 9, 75%). Patients with complicated appendicitis had a significantly higher rate of surgical complications (n = 8, 44.4%) compared to those without complicated appendicitis (n = 4, 2%, p = 0.000). Longer hospital stays were associated with a higher incidence of complications (p = 0.008). The American Society of Anesthesiologists classification showed a significant association with complications (p = 0.000).

Conclusions

Our study underscores the importance of early diagnosis, appropriate surgical management, and infection prevention strategies in reducing post-appendectomy complications. Factors such as complicated appendicitis, longer hospital stays, and higher ASA classification were associated with increased complication rates.

## Introduction

Abdominal pain is a common symptom encountered in clinical practice and can signify various underlying conditions, one of which is appendicitis [1]. Appendicitis is characterized by inflammation of the vermiform appendix and often manifests as acute abdominal pain, frequently necessitating emergency surgical intervention [2-4]. The incidence rate of appendicitis globally is approximately 8%, making it a prevalent condition that requires prompt diagnosis and management [5]. However, acute complicated appendicitis is a severe subset characterized by complications like perforation, abscess formation, or peritonitis, requiring urgent surgical intervention [1,2,4].

The standard treatment for acute appendicitis is appendectomy, a surgical procedure aimed at removing the inflamed appendix [3]. Appendectomy can be performed using either an open surgical approach or a laparoscopic method, each with its own unique set of advantages and potential risks [1]. Both surgical approaches for appendicitis are generally safe but carry risks of postoperative complications, including infections, abscesses, bowel obstructions, and deep vein thrombosis [4]. The reported complication rates following appendectomy range from 2% to 23%, highlighting the importance of assessing surgical outcomes and preventing adverse events [4]. One notable consideration in the choice of surgical approach is the duration of hospital stay, as open surgery is often associated with a longer postoperative recovery period compared to laparoscopy [4,5].

Approximately 3% of patients undergoing appendectomy may require readmission due to complications and the need for careful monitoring and follow-up care [3,4]. While some studies have reported no significant differences in postoperative complications between open and laparoscopic appendectomy methods [4], there remains a gap in the literature regarding comparative outcomes, especially in specific populations such as children and the elderly in Saudi Arabia [3]. Limited data on the effectiveness and safety of open versus laparoscopic appendectomy prompted the need for this retrospective study [2,5]. Although some studies have suggested that the timing of surgery (morning, afternoon, or night) does not significantly affect complication rates, there is a notable observation of increased appendicitis cases presenting at night, often managed with an open surgical approach [6,7]. Furthermore, comparisons between senior general surgeons (SGSs) and general surgery residents (GSR) have shown differences in surgical outcomes, with shorter surgery durations noted in SGSs [8].

The influence of factors such as the American Society of Anesthesiologists (ASA) classification on surgical outcomes, particularly in patients with comorbidities like asthma, has also been studied [9,10]. Additionally, laparoscopic appendectomy has been identified as a favorable option for obese patients due to shorter hospital stays [11]. Mortality rates post-appendectomy, especially in elderly patients with multiple comorbidities, have been documented, highlighting the importance of evaluating surgical safety and patient outcomes [12,13].

## Materials and methods

The study employed a retrospective research design aimed at assessing surgical outcomes of various surgical approaches for appendicitis in Taif City, Saudi Arabia. The study was conducted over a specific period from January 1, 2022, to December 31, 2022. The research was carried out at King Abdulaziz Specialist Hospital in Taif City, Saudi Arabia. This hospital was selected due to its status as a specialized medical center with a significant patient population, offering a suitable setting for studying appendectomy outcomes and surgical practices. The study included patients who underwent appendectomy during the specified study period of 2022. Both male and female patients across all age groups were considered eligible for inclusion, except for pregnant women who were excluded from the study due to unique physiological considerations related to pregnancy and surgery. A sample of 220 patients was included in the final analysis. The sampling approach aimed to include a diverse representation of patients undergoing both laparoscopic and open appendectomy. A structured data collection sheet was developed for this study (refer to Appendix A), comprising two main sections. The first section captured sociodemographic information such as age, gender, and preexisting conditions. The second section included variables related to the surgical procedure, complications, length of hospital stay, and postoperative outcomes. The data collection tool was adapted from previously validated instruments and tailored to the specific objectives of this study.

Data collection was conducted by reviewing medical files and electronic health records of patients who underwent appendectomy during the study period. Trained research personnel systematically extracted relevant data from patient charts, ensuring accuracy and consistency in data collection. The data collection process adhered to ethical guidelines and maintained patient confidentiality. Data processing and analysis were performed using IBM SPSS Statistics for Windows, Version 26.0 (Released 2019; IBM Corp., Armonk, NY, USA). Upon completion of data collection, the raw data were entered into the SPSS software for cleaning and organization. Data cleaning involved identifying and resolving any inconsistencies, missing values, or outliers to ensure the accuracy and reliability of the dataset. Descriptive statistics were used to summarize the demographic characteristics of the study participants, surgical procedures, and postoperative outcomes. Inferential statistical tests, including ANOVA, chi-square test, and Fisher’s exact test, were employed to analyze associations between variables and assess the significance of findings. A significance level of p < 0.05 was considered for all statistical tests. Ethical approval for this study was obtained from the Deanship of Scientific Research at the Ministry of Health in Taif City, Saudi Arabia (reference number HAP-02-T-067). The study adhered to ethical principles outlined in the Declaration of Helsinki, ensuring patient confidentiality, voluntary participation, and informed consent. Patient identities were anonymized in the data analysis process to protect privacy and confidentiality. Ethical considerations also included obtaining permissions for data access and ensuring compliance with institutional policies and regulations regarding research involving human subjects.

## Results

A total of 220 patients were included in the analysis, and their demographic characteristics, surgical details, and postoperative outcomes were examined. Table 1 presents a detailed overview of the patient’s demographics and surgical procedures. It is noted that the majority of patients fell into the age group of 20-39 years (n = 124, 56.4%), followed by those under 20 years (n = 68, 30.9%). In terms of gender distribution, 63.6% (n = 140) were male, and 36.4% (n = 80) were female. Regarding the presentation of appendicitis, 91.8% (n = 208) of patients did not present with complicated appendicitis, while 8.2% (n = 12) did. The most common type of surgery performed was open surgery, accounting for 86.4% (n = 190) of cases, while laparoscopic surgery was performed in 13.6% (n = 30) of cases.

**Table 1:** Characteristics of included patients and operations (n = 220) ASA, American Society of Anesthesiologists; PE, pulmonary embolism; SSI, surgical site infection

Parameters		Frequency (%)
Age (years)	<20	68 (30.9%)
	20-39	124 (56.4%)
	40-59	27 (12.3%)
	60-79	1 (0.5%)
Gender (female/male)	Female	80 (36.4%)
	Male	140 (63.6%)
Patient presented with complicated appendicitis	No	202 (91.8%)
	Yes	18 (8.2%)
Type of surgery	Laparoscopic	30 (13.6%)
	Open surgery	190 (86.4%)
Complicated surgery	No	208 (94.5%)
	Yes	12 (5.5%)
Type of complication (n = 12)	Hernia	2 (16.7%)
	PE	1 (8.3%)
	SSI	9 (75%)
Operation done by	Consultant	50 (22.7%)
	Resident	94 (42.7%)
	specialist	76 (34.5%)
Length of hospital stay (days)	One	50 (22.7%)
	Two	93 (42.3%)
	Three	45 (20.5%)
	Four	18 (8.2%)
	Five or more	14 (6.4%)
ASA	1	127 (57.7%)
	2	81 (36.8%)
	3	10 (4.5%)
	4	2 (0.9%)
Pre-perative antibiotic during hospital stay	No	52 (23.6%)
	Yes	168 (76.4%)
Time of performing the operation	Afternoon (15:00-22:00)	57 (25.9%)
	Morning (8:00-15:00)	78 (35.5%)
	Night (22:00-8:00)	85 (38.6%)

Complications post-surgery were observed in 5.5% (n = 12) of cases, with surgical site infection (SSI) being the most prevalent complication at 75% (n = 9). Other complications included hernia (n = 2, 16.7%) and pulmonary embolism (n = 1, 8.3%). The personnel performing the operations were mostly residents (n = 94, 42.7%) and specialists (n = 76, 34.5%), followed by consultants (n = 50, 22.7%). The length of hospital stay varied, with the majority of patients staying for two days (n = 93, 42.3%) or three days (n = 45, 20.5%). With regard to the time of performing the operation, 38.6% (n = 85) of the operations were performed in the night shift, 35.5% (n = 78) in the morning shift, and 25.9% (n = 57) in the afternoon shift.

Table 2 explores the association between surgical complications and various patient and operation characteristics. It was found that patients aged 40-59 years had a higher incidence of complicated surgeries (n = 4, 14.8%), although this difference was not statistically significant (p = 0.118). Similarly, gender did not show a significant association with complications (p = 0.145), although males tended to experience more complications (n = 10, 7.1%) than females (n = 2, 2.5%).

**Table 2:** Surgery complications in association with characters of patients and operations (n = 220) ASA, American Society of Anesthesiologists

Parameters		Complicated surgery		X^2^	p-value
		No	Yes		
Age (years)	<20	64 (94.1%)	4 (5.9%)	5.863	0.118
	20-39	120 (96.8%)	4 (3.2%)		
	40-59	23 (85.2%)	4 (14.8%)		
	60-79	1 (100%)	0 (0%)		
Gender (female/male)	Female	78 (97.5%)	2 (2.5%)	2.128	0.145
	Male	130 (92.9%)	10 (7.1%)		
Patient presented with complicated appendicitis	No	198 (98%)	4 (2%)	57.789	0
	Yes	10 (55.6%)	8 (44.4%)		
Type of surgery	Laparoscopic	29 (96.7%)	1 (3.3%)	0.303	0.582
	Open surgery	179 (94.2%)	11 (5.8%)		
Operation done by	Consultant	48 (96%)	2 (4%)	7.884	0.061
	Resident	91 (96.8%)	3 (3.2%)		
	Specialist	69 (87.7%)	7 (12.3%)		
Length of hospital stay (days)	One	50 (100%)	0 (0%)	13.866	0.008
	Two	87 (93.5%)	6 (6.5%)		
	Three	44 (97.8%)	1 (2.2%)		
	Four	14 (77.8%)	4 (22.2%)		
	Five or more	13 (92.9%)	1 (7.1%)		
ASA	1	126 (99.2%)	1 (0.8%)	63.253	0
	2	76 (93.8%)	5 (6.2%)		
	3	4 (40%)	6 (60%)		
	4	2 (100%)	0 (0%)		
Preoperative antibiotic during hospital stay	No	49 (94.2%)	3 (5.8%)	0.013	0.909
	Yes	159 (94.6%)	9 (5.4%)		
Time of performing the operation	Afternoon (15:00-22:00)	54 (94.7%)	3 (5.3%)	2.542	0.281
	Morning (8:00-15:00)	76 (97.4%)	2 (2.6%)		
	Night (22:00-8:00)	78 (91.8%)	7 (8.2%)		

The presentation of complicated appendicitis was significantly associated with a higher rate of surgical complications (n = 8, 44.4% vs. n = 4, 2%, p = 0.000). Additionally, the length of hospital stay showed a significant association with complications (p = 0.008), with longer stays correlating with a higher incidence of complications. The ASA classification also demonstrated a significant association with complications (p = 0.000), as higher ASA scores were linked to increased rates of surgical complications.

However, factors such as the type of surgery, operating personnel, preoperative antibiotic use, and time of performing the operation did not show significant associations with surgical complications (p > 0.05).

**Figure 1:**
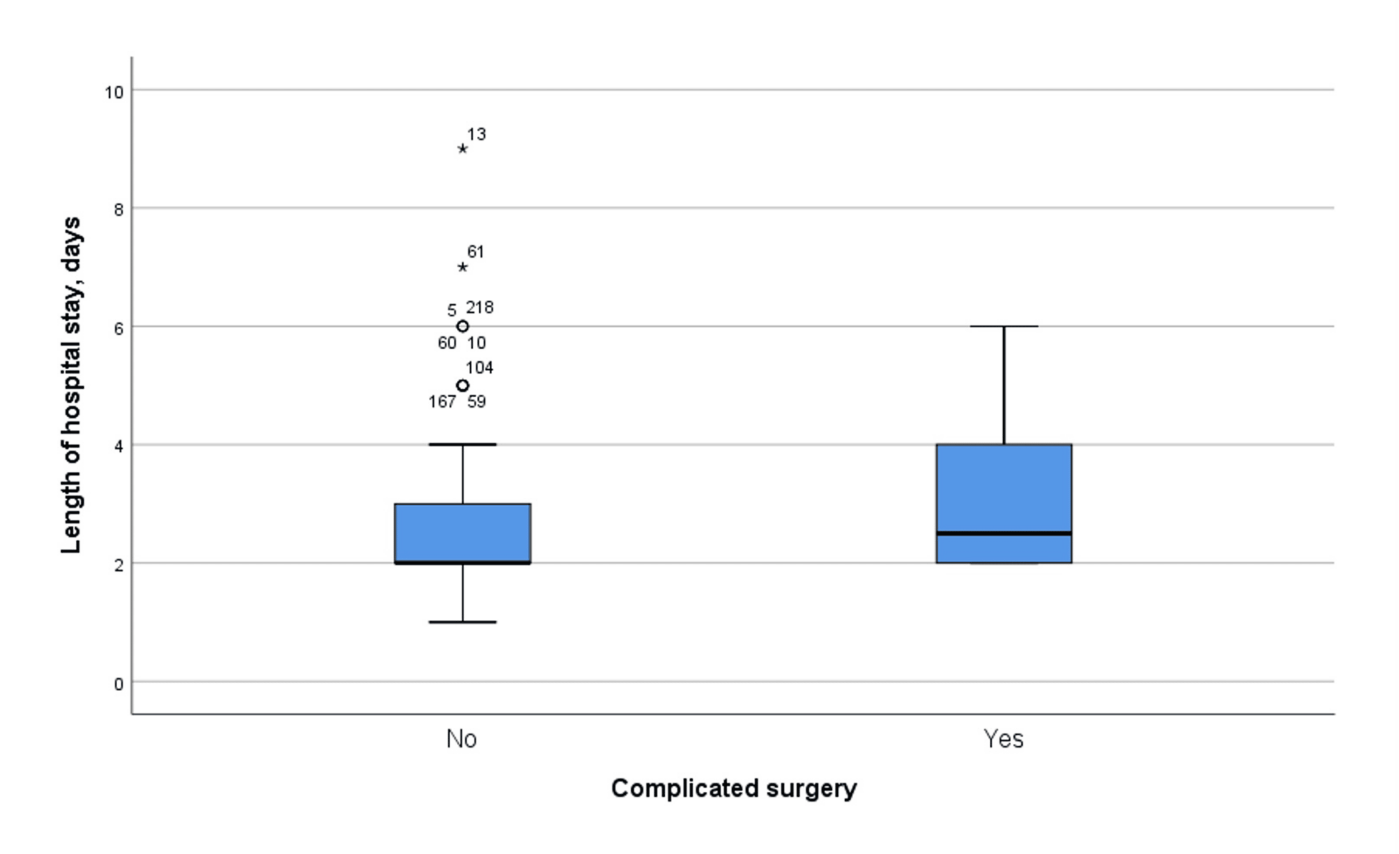
Boxplot showing average hospital stay (days) among complicated and non-complicated groups (F = 3.939, p = 0.048)

Figure 1 presents the average hospital stay (in days) among patients categorized into complicated and non-complicated groups following appendectomy. The analysis revealed a significant difference between these groups (F = 3.939, p = 0.048), indicating that patients with complications had a longer average hospital stay compared to those without complications.

Table 3 delves into the specific types of complications observed and their association with patient and operation characteristics. Among the types of complications (hernia, PE, and SSI), SSI was the most prevalent (n = 9, 75%).

**Table 3:** Type of complication in association with characters of patients and operations (n = 12) ASA, American Society of Anesthesiologists; PE, pulmonary embolism; SSI, surgical site infection

Parameters		Type of complication (n = 12)			X^2^	p-value
		Hernia	PE	SSI		
Age (years)	<20	0 (0%)	1 (25%)	3 (75%)	3	0.558
	20-39	1 (25%)	0 (0%)	3 (75%)		
	40-59	1 (25%)	0 (0%)	3 (75%)		
	60-79	0 (0%)	0 (0%)	0 (0%)		
Gender (female/male)	Female	0 (0%)	1 (50%)	1 (50%)	5.6	0.061
	Male	2 (20%)	0 (0%)	8 (80%)		
Patient presented with complicated appendicitis	No	0 (0%)	1 (25%)	3 (75%)	3	0.223
	Yes	2 (25%)	0 (0%)	6 (75%)		
Type of surgery	Laparoscopic	0 (0%)	0 (0%)	1 (100%)	0.364	0.834
	Open surgery	2 (18.2%)	1 (9.1%)	8 (72.7%)		
Operation done by	Consultant	1 (50%)	0 (0%)	1 (50%)	5.159	0.271
	Resident	0 (0%)	1 (33.3%)	2 (66.7%)		
	Specialist	1 (14.3%)	0 (0%)	6 (85.7%)		
Length of hospital stay (days)	One	0 (0%)	0 (0%)	0 (0%)	18.5	0.005
	Two	0 (0%)	0 (0%)	6 (100%)		
	Three	1 (100%)	0 (0%)	0 (0%)		
	Four	1 (25%)	0 (0%)	3 (75%)		
	Five or more	0 (0%)	1 (100%)	0 (0%)		
ASA	1	0 (0%)	0 (0%)	1 (100%)	3.556	0.469
	2	0 (0%)	1 (20%)	4 (80%)		
	3	2 (33.3%)	0 (0%)	4 (66.7%)		
	4	0 (0%)	0 (0%)	0 (0%)		
Preoperative antibiotic during hospital stay	No	1 (33.3%)	0 (0%)	2 (66.7%)	1.037	0.595
	Yes	1 (11.1%)	1 (11.1%)	7 (77.8%)		
Time of performing the operation	Afternoon (15:00-22:00)	1 (33.3%)	0 (0%)	2 (66.7%)	6.159	0.188
	Morning (8:00-15:00)	0 (0%)	1 (50%)	1 (50%)		
	Night (22:00-8:00)	1 (14.3%)	0 (0%)	6 (85.7%)		

While age groups and gender did not show significant associations with specific complications (p > 0.05), patients presenting with complicated appendicitis had a higher proportion of complications, particularly SSI (n = 6, 75%) compared to those SSI cases without complicated appendicitis (n = 3, 25%, p = 0.223). The length of hospital stay was significantly associated with the type of complication (p = 0.005), with longer stays correlating with a higher incidence of SSI. Time of performing the operation was insignificantly associated with the type of complication (p =0.188).

## Discussion

Appendicitis remains a common surgical emergency worldwide, requiring prompt diagnosis and appropriate management to prevent complications and improve patient outcomes [1,2]. The definitive treatment for appendicitis is an appendectomy, which can be performed using open surgical techniques or laparoscopy [3,14]. While laparoscopic appendectomy has gained popularity due to its minimally invasive nature and potential for reduced postoperative complications, open surgery remains a viable option, especially in cases of complicated appendicitis or in settings where laparoscopic expertise may be limited [3-6]. Our study aimed to assess surgical outcomes of various surgical approaches for appendicitis in Taif City, Saudi Arabia. We conducted a retrospective analysis of 220 patients who underwent appendectomy at King Abdulaziz Specialist Hospital in 2022. Our main findings revealed several key insights into the demographic characteristics, surgical procedures, and postoperative outcomes of these patients.

Most patients did not present with complicated appendicitis, and open surgery was the preferred surgical approach. Complications post-surgery were observed in a small percentage of cases, with SSI being the most common complication. A higher incidence of complications was associated with longer hospital stays, and patients with complicated appendicitis had a significantly higher rate of surgical complications compared to those without complicated appendicitis [15-17]. One of the primary objectives of our study was to assess the effectiveness and safety of laparoscopic versus open appendectomy. Our results indicated that the majority of appendectomies in Taif City were performed using open surgery (n = 190, 86.4%), with laparoscopic surgery accounting for a smaller percentage (n = 30, 13.6%). This distribution reflects common practice patterns in many healthcare settings, where the choice of surgical approach may depend on various factors such as surgeon expertise, patient characteristics, and hospital resources [2-4].

The literature provides mixed evidence regarding the superiority of laparoscopic appendectomy over open surgery [18-20]. This preference may be influenced by several factors including surgeon expertise, patient characteristics, and hospital resources [3]. While laparoscopy is associated with shorter hospital stays and reduced wound complications, some studies have not found significant differences in overall postoperative complications between the two methods [4]. Contrary to some literature suggesting superior outcomes with laparoscopic appendectomy [4], we did not find a significant association between the type of surgery and overall postoperative complications. Our results align with studies that have reported similar complication rates between open and laparoscopic approaches (p > 0.05) [4,18]. However, it is crucial to note that individual patient factors and case complexity may influence the choice of surgical approach and subsequent outcomes.

Complicated appendicitis poses a greater challenge in terms of surgical management and postoperative care [21,22]. In our study, patients presenting with complicated appendicitis had a significantly higher rate of surgical complications (n = 8, 44.4%) compared to those without complications (n = 4, 2%, p = 0.000). This finding underscores the importance of early diagnosis and intervention to prevent appendiceal perforation and subsequent complications [21,23]. The literature consistently supports the notion that complicated appendicitis is associated with increased morbidity, longer hospital stays, and higher rates of postoperative complications such as SSIs and intra-abdominal abscesses [2,5]. Our study adds to this body of evidence by highlighting the impact of complicated appendicitis on surgical outcomes in the Taif City population.

Several other factors were explored in our study to understand their potential influence on post-appendectomy complications. The ASA classification showed a significant association with complications (p = 0.000). Higher ASA scores were correlated with increased rates of surgical complications, emphasizing the importance of preoperative risk stratification and optimization of patient health status [13]. The timing of appendectomy, whether performed during morning, afternoon, or night, did not significantly affect complication rates as reported in the literature [21,22]. This is consistent with studies indicating that the timing of surgery does not necessarily impact postoperative outcomes, although the volume of appendectomies may vary throughout the day [6].

Furthermore, our study did not find significant differences in complication rates between surgeries performed by SGSs and GSRs, contrary to some previous studies suggesting that experienced surgeons may have better outcomes [8]. However, it is essential to note that surgical expertise and skill levels can vary widely among practitioners, potentially influencing complication rates. Comparing our findings with international studies, we observed similar trends regarding the impact of complicated appendicitis on postoperative complications and the lack of significant differences between laparoscopic and open surgery in terms of overall complication rates. Studies from Australia [12] and the United States [10] have also highlighted the importance of patient risk stratification, surgical expertise, and standardized protocols in appendicitis management.

Strengths and limitations

The study examined the effectiveness and safety of open versus laparoscopic appendectomy and compared outcomes between senior surgeons and residents, noting that most procedures are performed by residents. It also considered the impact of the ASA classification on outcomes. Limitations include relying solely on medical file data, suggesting follow-up studies to track complications over time, and focusing on 220 patients in 2022 without historical comparisons or control groups to adequately assess risks.

## Conclusions

Our study compared open and laparoscopic appendectomy outcomes in Taif City, Saudi Arabia. Higher ASA classifications correlated with increased post-appendectomy complications. These findings emphasize the importance of tailored surgical strategies, preoperative risk assessment, and continuous monitoring to optimize appendectomy outcomes and patient care. Future research should focus on long-term follow-up and prospective studies to further refine surgical practices and enhance patient outcomes.

## Appendices

Appendix A

Data collected from files according to these questions

1. Age group

Less than 20 years old

20-39

40-59

60-79

2. Gender

Male

Female

3. Patient came with complicated appendicitis

Yes

No

4. Type of surgery to treat appendicitis

Open appendectomy

Laparoscopic appendectomy

5. Is there any complication after surgery?

Yes

No

6. Type of complication

SSI

PE

Hernia

7. Operation done by

Consultant

Resident

Specialist

8. Length of hospital stay

One day

Two days

Three days

Four days

Five or more

9. American Society of Anesthesiologists (ASA)

1

2

3

4

10. Preoperative antibiotic during hospital stay

Yes

No

11. Time of performing the operation

Morning (8:00-15:00)

Afternoon (15:00-22:00)

Night (22:00-8:00)
